# Commentary on: The Utilization of Poly-l-Lactic Acid as a Safe and Reliable Method for Volume Maintenance After Facelift Surgery With Fat Grafting

**DOI:** 10.1093/asjof/ojac022

**Published:** 2022-04-21

**Authors:** Gabriele Cáceres Miotto

The authors present a retrospective review of 241 patients who underwent facelift surgery with fat grafting, associated with Poly-L-Lactic Acid (PLLA) injections for facial volume maintenance after surgery.^1^ They selected PLLA as the main filler used reporting long-term stability of volume replacement - up to four years - in patients with no weight fluctuations. The authors had 5 indications for PLLA injections and used subjective before and after photographs to evaluate its effectiveness. It would have been powerful to see a more objective assessment of volume retention such as three-dimensional imaging or patient reported outcome metrics using validated survey instruments (Video). 

An important detail of this study was that the authors used an off-label dilution of PLLA, a common practice in facial aesthetics, reporting no complications or nodule formation in this series. Integrating facial fillers into postsurgical maintenance regime is especially helpful in patients with very low body mass index and inadequate fat donor sites. This paper reflects current clinical facial aesthetic surgery practices on facial volumization. In addition to PLLA, in our practice and many others, the use of different filler types such as hyaluronic acid (HA) and calcium hydroxyapatite is also very helpful for ongoing facial volumization and rejuvenation.
